# Association of Postoperative Undertriage to Hospital Wards With Mortality and Morbidity

**DOI:** 10.1001/jamanetworkopen.2021.31669

**Published:** 2021-11-10

**Authors:** Tyler J. Loftus, Matthew M. Ruppert, Tezcan Ozrazgat-Baslanti, Jeremy A. Balch, Philip A. Efron, Patrick J. Tighe, William R. Hogan, Parisa Rashidi, Gilbert R. Upchurch, Azra Bihorac

**Affiliations:** 1 Department of Surgery, University of Florida Health, Gainesville; 2 Precision and Intelligent Systems in Medicine Research Partnership, University of Florida, Gainesville; 3 Department of Medicine, University of Florida Health, Gainesville; 4 Department of Anesthesiology, University of Florida Health, Gainesville; 5 Department of Orthopaedic Surgery and Sports Medicine, University of Florida Health, Gainesville; 6 Department of Information Systems and Operations Management, University of Florida Health, Gainesville; 7 Department of Health Outcomes and Biomedical Informatics, University of Florida College of Medicine, Gainesville; 8 Department of Biomedical Engineering, University of Florida, Gainesville; 9 Department of Computer and Information Science and Engineering, University of Florida, Gainesville; 10 Department of Electrical and Computer Engineering, University of Florida, Gainesville

## Abstract

**Importance:**

Undertriaging patients who are at increased risk for postoperative complications after surgical procedures to low-acuity hospital wards (ie, floors) rather than highly vigilant intensive care units (ICUs) may be associated with risk of unrecognized decompensation and worse patient outcomes, but evidence for these associations is lacking.

**Objective:**

To test the hypothesis that postoperative undertriage is associated with increased mortality and morbidity compared with risk-matched ICU admission.

**Design, Setting, and Participants:**

This longitudinal cross-sectional study was conducted using data from the University of Florida Integrated Data Repository on admissions to a university hospital. Included patients were individuals aged 18 years or older who were admitted after a surgical procedure from June 1, 2014, to August 20, 2020. Data were analyzed from April through August 2021.

**Exposures:**

Ward admissions were considered undertriaged if their estimated risk for hospital mortality or prolonged ICU stay (ie, ≥48 hours) was in the top quartile among all inpatient surgical procedures according to a validated machine-learning model using preoperative and intraoperative electronic health record features available at surgical procedure end time. A nearest neighbors algorithm was used to identify a risk-matched control group of ICU admissions.

**Main Outcomes and Measures:**

The primary outcomes of hospital mortality and morbidity were compared among appropriately triaged ward admissions, undertriaged wards admissions, and a risk-matched control group of ICU admissions.

**Results:**

Among 12 348 postoperative ward admissions, 11 042 admissions (89.4%) were appropriately triaged (5927 [53.7%] women; median [IQR] age, 59 [44-70] years) and 1306 admissions (10.6%) were undertriaged and matched with a control group of 2452 ICU admissions. The undertriaged group, compared with the control group, had increased median [IQR] age (64 [54-74] years vs 62 [50-73] years; *P* = .001) and increased proportions of women (649 [49.7%] women vs 1080 [44.0%] women; *P* < .001) and admitted patients with do not resuscitate orders before first surgical procedure (53 admissions [4.1%] vs 27 admissions [1.1%]); *P* < .001); 207 admissions that were undertriaged (15.8%) had subsequent ICU admission. In the validation cohort, hospital mortality and prolonged ICU stay estimations had areas under the receiver operating characteristic curve of 0.92 (95% CI, 0.91-0.93) and 0.92 (95% CI, 0.92-0.92), respectively. The undertriaged group, compared with the control group, had similar incidence of prolonged mechanical ventilation (32 admissions [2.5%] vs 53 admissions [2.2%]; *P* = .60), decreased median (IQR) total costs for admission ($26 900 [$18 400-$42 300] vs $32 700 [$22 700-$48 500]; *P* < .001), increased median (IQR) hospital length of stay (8.1 [5.1-13.6] days vs 6.0 [3.3-9.3] days, *P* < .001), and increased incidence of hospital mortality (19 admissions [1.5%] vs 17 admissions [0.7%]; *P* = .04), discharge to hospice (23 admissions [1.8%] vs 14 admissions [0.6%]; *P* < .001), unplanned intubation (45 admissions [3.4%] vs 49 admissions [2.0%]; *P* = .01), and acute kidney injury (341 admissions [26.1%] vs 477 admissions [19.5%]; *P* < .001).

**Conclusions and Relevance:**

This study found that admitted patients at increased risk for postoperative complications who were undertriaged to hospital wards had increased mortality and morbidity compared with a risk-matched control group of admissions to ICUs. Postoperative undertriage was identifiable using automated preoperative and intraoperative data as features in real-time machine-learning models.

## Key Points

**Question:**

Is postoperative undertriage associated with increased mortality, morbidity, and resource use?

**Findings:**

In this cross-sectional study of 14 890 postoperative admissions, undertriage to hospital wards was associated with increased mortality and morbidity compared with admissions that had similar risk profiles and were triaged to intensive care units. Postoperative undertriage was identifiable using automated preoperative and intraoperative data as features for real-time machine-learning models.

**Meaning:**

These findings suggest that there is a rationale and framework for clinical decision support platforms to augment postoperative triage decisions.

## Teaser

This cross-sectional study investigates the association of undertriage to hospital wards after surgical procedures with mortality, morbidity, and resource use.

## Introduction

Approximately 15 million major inpatient surgical procedures are performed in the United States annually.^1^ Immediately after surgical procedures, surgeons must determine whether patients can be triaged safely to hospital wards (ie, floors) with high patient-to-clinician ratios and infrequent assessments. When patients with high acuity are undertriaged to hospital wards, postoperative complications may progress to critical illness and cardiac arrest between patient assessments that are typically spaced every 4 hours.^2,3,4,5,6^ Approximately 41% of hospital arrests occur on wards, at 0.1 per 1000 bed-days, with 80% to 85% hospital mortality.^4,6,7^ The underlying disease process is often reversible, suggesting opportunities for prevention with appropriate surveillance and early intervention.^8^

Despite the intuitive importance of undertriage, there is a paucity of evidence regarding associations between postoperative undertriage and patient outcomes. Among hospital admissions involving surgical procedures in the United Kingdom, 85% of all patients undergoing high-risk procedures (ie, those with mortality rates ≥5%) were admitted to hospital wards; patients with subsequent intensive care unit (ICU) admission had a 37% incidence of mortality.^2^ In a similar analysis, 74% of postoperative deaths occurred outside the ICU.^3^ These findings are compelling, but they use procedure-associated mortality alone to identify patients at increased risk. Granular, patient-level acuity assessments may further elucidate the sequelae associated with postoperative undertriage.

Using a longitudinal cohort of postoperative admissions, we tested the hypothesis that postoperative undertriage would be associated with increased mortality and morbidity compared with risk-matched ICU admission. Undertriaged ward admissions were identified by a validated machine-learning model using automated electronic health record (EHR) data to generate risk estimations at surgical procedure end time, when triage decisions were finalized.^9,10^ We compared mortality and morbidity among appropriately triaged ward admissions, undertriaged wards admissions, and risk-matched ICU admissions.

## Methods

The University of Florida Institutional Review Board approved this cross-sectional study with full waiver of informed consent with the justification that this retrospective study involved no interventions and it would be difficult to contact thousands patients who received care many years prior to enrollment. This study complies with the Strengthening the Reporting of Observational Studies in Epidemiology (STROBE) reporting guideline.

### Study Design

We generated a longitudinal cohort of patients aged 18 years or older undergoing inpatient surgical procedures at a university hospital. Ward admissions were considered undertriaged if their estimated risk for hospital mortality or prolonged ICU stay (ie, ≥48 hours) was in the top quartile among inpatient surgical procedures according to a validated machine-learning model using preoperative and intraoperative estimator features extracted directly from EHRs, generating estimations at surgical procedure end time; risk thresholds were applied to these estimations.^9,10^ A nearest neighbors algorithm was used to identify a risk-matched control ICU admissions. Matching was performed on composite risk for hospital mortality or prolonged ICU stay represented by the mean *z* score across both outcomes. The primary analysis compared mortality and morbidity among admissions that were appropriately triaged to hospital wards or undertriaged to hospital wards and members of a risk-matched control group.

### Data Source

The University of Florida Integrated Data Repository functioned as honest broker in assembling the study cohort, consisting of 71 065 hospital admissions for inpatient surgical procedures at University of Florida Health in Gainesville, Florida, from June 1, 2014, to August 20, 2020. Derivation of the study population is illustrated in eFigure 1 in the Supplement. Briefly, we included inpatient surgical admissions and excluded organ donation surgical procedures, anesthesia outside the operating room, and admissions lasting less than 24 hours, which are often considered observation only, failing to represent major inpatient surgical procedures. Deaths within 24 hours were excluded to avoid excessively narrow estimation windows. The study cohort was split chronologically into development and validation cohorts to allow nonrandom variation between data sets and mitigate potentially adverse outcomes associated with data set drift for algorithm estimations, consistent with Transparent Reporting of a Multivariable Prediction Model for Individual Prognosis or Diagnosis (TRIPOD) reporting guideline recommendations under the type 2b analysis category.^11^ The final data set included granular information from preoperative, intraoperative, and postoperative phases of care.^9,10^ Descriptions of all data elements and their preprocessing steps are provided in eMethods in the Supplement.

### Model Development and Validation

The model used a data transformer layer to perform preprocessing, feature transformation, and feature selection techniques.^9,10^ A data analytics layer computed separate, patient admission–specific risk probabilities for hospital mortality and prolonged ICU stay using a random forest algorithm.^10^ The model was trained on a development cohort of 49 687 postoperative admissions, using only preoperative and intraoperative data as input features. The trained model made estimations on 20 940 validation cohort admissions; results are reported exclusively from this separate cohort.

### Identifying Undertriaged Ward Admissions

In identifying undertriage, the top quartile (ie, ≥75th percentile) cutoff for hospital mortality risk was chosen because it corresponds to 5% incidence of mortality, consistent with definitions of high-risk surgical procedures in peer-reviewed literature.^2,3,12^ Regarding risk of prolonged ICU stay, for which there is no precedent in peer-reviewed literature, the top quartile cutoff was chosen to maintain consistency with the mortality cutoff.

### Identifying Risk-Matched Control ICU Admissions

To identify a control population of postoperative ICU admissions, we fit a nearest neighbors, brute-force algorithm^13^ on estimations for hospital mortality and prolonged ICU stay among ICU admissions that did not receive immediate postoperative mechanical ventilation or vasopressor support (ie, within 2 hours of surgical procedure end time) and applied the fit model to the undertriaged group using a maximum Minkowski distance of 1 to identify the 3 nearest neighbors for each admission. Matching was performed on composite risk for hospital mortality or prolonged ICU stay represented by the mean *z* score across these outcomes. This method matched 2452 control admissions with 1306 undertriage admissions.

### Hospital Stations

We extracted EHR-embedded hospital station labels with entrance and exit date times. Level of care assignments allowed ICU admission under intensive status, requiring patient-to-nurse ratios of 2:1 or less, or intermediate status, requiring patient-to-nurse ratios of 3:1 or less. The ICU was considered as a single destination that provided low patient-to-nurse ratios and consistent availability of critical care resources and personnel.

### Statistical Analysis

We performed a power analysis using data from a peer-reviewed study of 121 290 inpatient surgical procedures.^14^ Planned surgical complexity grade 4 was observed among 35 931 hospital admissions and was associated with hospital mortality among 1863 admissions (5.18%), corresponding to high-risk surgical procedures definitions.^2,3,12^ Planned surgical complexity grade 1 was observed among 13 484 hospital admissions and was associated with hospital mortality among 49 admissions (0.36%). Accounting for imbalanced cohorts and hospital mortality as low as among 16 admissions (1.24%) in the high-risk group, our sample would detect a difference in hospital mortality of 1.19 percentage points, with 80% power and α = .05. Other outcomes reported were exploratory. While conducting this study, we observed that the undertriaged group had increased area deprivation indices and increased proportions of patients identifying as Black or African American and with preoperative do not resuscitate (DNR) orders; we performed exploratory, post hoc, secondary analyses for these 3 variables. Race data were collected from electronic health records using self-reported classifications. Race was assessed to determine whether there were associations among race, postoperative triage decisions, and patient outcomes. All primary outcome analyses were adjusted for multiple comparisons using the Benjamini-Hochberg procedure.

For model training, missing values were replaced with a distinct missing indicator; for reporting results, missing values were imputed with medians. Continuous variables were reported as median values with IQR and compared by the Kruskal-Wallis test. Discrete variables were reported as raw numbers with percentages and compared by Fisher exact test. Model performance was assessed by calculating area under the receiver operating characteristic curves (AUROCs) and area under the precision recall curves (AUPRCs) with 95% CIs. All hypothesis tests were 2-sided with α = .05. The power analysis was performed using PASS statistical software (NCSS). Other statistical analyses were performed with Python programming language version 3.8.8 (Python Software Foundation). Data were analyzed from April through August 2021.

## Results

### Study Population Characteristics

Patient characteristics are summarized in Table 1. Among 12 348 postoperative ward admissions, 11 042 admissions (89.4%) were appropriately triaged and 1306 admissions (10.6%) were undertriaged and matched with a control group of 2452 ICU admissions. The undertriaged group, compared with the appropriately triaged group, had a decreased proportion of women (649 [49.7%] women vs 5927 [53.7%] women; *P* = .007), increased median (IQR) age (64 [54-74] vs 59 [44-70] years; *P* < .001), and an increased proportion of admitted patients self-identifying as Black or African American (261 admissions [20.0%] vs 1627 admission [14.7%]; *P* < .001), with a decreased proportion of admitted patients self-identifying as White (956 admissions [73.2%] vs 8483 admissions [76.8%]; *P* = .004) and a similar proportion of admitted patients self-identifying as Hispanic (2 admissions [0.2%] vs 20 admissions [0.2%]; *P* > .99). Area deprivation indices were increased among undertriaged admissions, suggesting socioeconomic vulnerability. The undertriaged group had increased median [IQR] Charlson Comorbidity Index score (2.0 [1.0-4.0] vs 1.0 [0-2.0]; *P* < .001) and increased proportions of admitted patients undergoing emergent admission (868 admissions [66.5%] vs 3369 admissions [30.5%]; *P* < .001), preoperative red blood cell transfusion (87 admissions [6.7%] vs 84 admissions [0.8%]; *P* < .001), and emergent surgical procedures (298 admissions [22.8%] vs 1352 admissions [12.2%]; *P* < .001).

**Table 1.** Patient Characteristics

Characteristic	Admissions, No. (%)			*P* value^a^	
	Appropriate triage (n = 11 042)	Undertriage (n = 1306)	Control (n = 2452)	Appropriate triage vs undertriage	Undertriage vs control
Demographics					
Women	5927 (53.7)	649 (49.7)	1080 (44.0)	.007	<.001
Men	5115 (46.3)	657 (50.3)	1372 (56.0)		
Age, median (IQR), y	59.0 (44.0-70.0)	64.0 (54.0-74.0)	62.0 (50.0-73.0)	<.001	.001
Race					
American Indian or Alaska Native	17 (0.2)	0	5 (0.2)	.25	.17
Asian	101 (0.9)	9 (0.7)	20 (0.8)	.53	.85
Black or African American	1627 (14.7)	261 (20.0)	331 (13.5)	<.001	<.001
Hispanic	20 (0.2)	2 (0.2)	2 (0.1)	>.99	.61
Native Hawaiian or other Pacific Islander	2 (<0.1)	0	0	>.99	>.99
White	8483 (76.8)	956 (73.2)	1946 (79.4)	.004	<.001
Other^b^ or multiracial	605 (5.5)	63 (4.8)	113 (4.6)	.37	.81
Unknown	187 (1.7)	15 (1.1)	35 (1.4)	.17	.55
ADI score, median (IQR)					
State rank	7.0 (4.0-9.0)	8.0 (5.0-9.0)	7.0 (5.0-9.0)	<.001	.004
National rank	69.0 (46.0-85.0)	73.0 (53.0-85.0)	68.0 (47.0-85.0)	<.001	.004
Illness severity, median (IQR)					
ASA score	3.0 (2.0-3.0)	3.0 (3.0-3.0)	3.0 (3.0-3.0)	<.001	.16
Charlson Comorbidity Index score	1.0 (0-2.0)	2.0 (1.0-4.0)	2.0 (0-3.0)	<.001	<.001
SOFA score	0 (0-0)	0 (0-0)	1.0 (0-2.0)	<.001	<.001
Admitted from emergency department	3319 (30.1)	810 (62.0)	850 (34.7)	<.001	<.001
Red blood cell transfusion					
Preoperative	84 (0.8)	87 (6.7)	68 (2.8)	<.001	<.001
Intraoperative	23 (0.2)	5 (0.4)	13 (0.5)	.21	.63
Admission priority					
Elective	7158 (64.8)	305 (23.4)	1354 (55.2)	<.001	<.001
Urgent	130 (1.2)	49 (3.8)	64 (2.6)	<.001	.06
Emergent	3369 (30.5)	868 (66.5)	848 (34.6)	<.001	<.001
Trauma activation or transfer	383 (3.5)	84 (6.4)	186 (7.6)	<.001	.21
Unknown	2 (<0.1)	0	0	>.99	>.99
Surgical procedure priority					
Elective	9070 (82.1)	920 (70.4)	1919 (78.3)	<.001	<.001
Urgent	620 (5.6)	88 (6.7)	89 (3.6)	.10	<.001
Emergent	1352 (12.2)	298 (22.8)	444 (18.1)	<.001	<.001

Abbreviations: ADI, area deprivation index; ASA, American Society of Anesthesiologists; SOFA, Sequential Organ Failure Assessment.

^a^ *P* values are from significance tests comparing appropriate triage, undertriage, and control groups by each variable.

^b^ Other was a category for self-reporting race that patients could select.

The undertriaged group, compared with the control group, had increased median [IQR] age (vs 62 [50-73] years; *P* = .001) and increased proportions of women (vs 1080 [44.0%] women; *P* < .001) and admitted patients self-identifying as Black or African American (vs 331 admissions [13.5%]; *P* < .001), with a decreased proportion of admitted patients self-identifying as White (vs 1946 admissions [79.4%]; *P* < .001) and a similar proportion of admitted patients self-identifying as Hispanic (vs 2 admissions [0.1%]; *P* = .61). Area deprivation indices were increased among undertriaged admissions compared with admissions in the control group. American Society of Anesthesiologists class distributions were similar between these 2 groups, but the undertriaged group had an increased median (IQR) Charlson Comorbidity Index score (vs 2.0 [0-3.0]; *P* < .001) and decreased median (IQR) Sequential Organ Failure Assessment score on admission (0 [0-0] vs 1.0 [0-2.0]; *P* < .001). The undertriaged group had increased proportions of admitted patients undergoing emergent admission (vs 848 admissions [34.6%]; *P* < .001) and emergent surgical procedures (vs 444 admissions [18.1%]; *P* < .001) and with preoperative red blood cell transfusions (vs 68 admissions [2.8%]; *P* < .001). Primary surgical services for each admission and service-specific mortality rates are listed in eTable 1 and eTable 2 in the Supplement, respectively.

### Triage Classifications

In the validation cohort, hospital mortality estimations had an AUROC of 0.92 (95% CI, 0.91-0.93) and an AUPRC of 0.26 (95% CI, 0.23-0.30); prolonged ICU stay estimations had an AUROC of 0.92 (95% CI, 0.92-0.92) and an AUPRC of 0.85 (95% CI, 0.85-0.86). According to these estimations, 11 042 postoperative ward admissions were appropriate and 1306 admissions were undertriaged, representing 10.6% of all ward admissions. The 20 most important features (as defined by weight, which was derived by calculating change in node impurity and the probability of reaching each node) for estimating hospital mortality and prolonged ICU stay are listed in eTable 3 and eTable 4 in the Supplement, respectively. Primary procedure, scheduled postoperative location, and intraoperative minimum alveolar concentration measurements or inhalational anesthetic duration were among the 5 most important features for both estimations.

### Perioperative Factors

Perioperative factors are summarized in Table 2. Increased proportions of undertriaged admissions, compared with appropriately triaged admissions, were planned for postoperative ICU admission under intensive status (91 admissions [7.0%] vs 284 admissions [2.6%]; *P* < .001) and intermediate status (19 admissions [1.5%] vs 66 admissions [0.6%]; *P* = .002). Immediately after the surgical procedure, undertriaged admissions had increased median (IQR) heart rate (82.1 beats per minute [bpm; 72.2-91.4 bpm] vs 79.8 bpm [71.2-89.0 bpm]; *P* < .001) and decreased median (IQR) systolic blood pressure (124.3 [111.0-138.9] mm Hg vs 126.1 [114.5-138.0] mm Hg; *P* < .001) but similar respiratory rate, oxygen saturation, and Glasgow coma scale eye opening response. An increased proportion of undertriaged admissions had cardiac telemetry ordered prior to ward admission (225 admissions [17.2%] vs 460 admissions [4.2%]; *P* < .001) compared with appropriately triaged admissions, while the proportion with a continuous pulse oximetry order was similar between these 2 groups (1094 admissions [83.8%] vs 9408 admissions [85.2%]; *P* = .18). The undertriaged group had an increased proportion of admitted patients with DNR orders placed preoperatively (53 admissions [4.1%] vs 55 admissions [0.5%]; *P* < .001) and during admission (91 admissions [7.0%] vs 78 admissions [0.7%]; *P* < .001) compared with the appropriately triaged group.

**Table 2.** Admitted Patient Perioperative Factors

Perioperative factor^b^	Admissions, No. (%)			*P* value^a^	
	Appropriate triage (n = 11 042)	Undertriage (n = 1306)	Control (n = 2452)	Appropriate triage vs undertriage	Undertriage vs control
Planned postoperative destination					
ICU, intensive status	284 (2.6)	91 (7.0)	1570 (64.0)	<.001	<.001
ICU, intermediate status	66 (0.6)	19 (1.5)	237 (9.7)	.002	<.001
Postanesthesia care unit and ward	10 665 (96.6)	1173 (89.8)	630 (25.7)	<.001	<.001
Unknown	27 (0.2)	23 (1.8)	15 (0.6)	<.001	.002
Immediate postoperative vital signs, median (IQR)^c^					
Heart rate, bpm	79.8 (71.2-89.0)	82.1 (72.2-91.4)	80.5 (70.2-91.2)	<.001	.01
Blood pressure, mm Hg					
Systolic	126.1 (114.5-138.0)	124.3 (111.0-138.9)	131.0 (116.4-145.4)	<.001	<.001
Diastolic	70.3 (62.8-78.0)	66.4 (58.2-74.8)	67.0 (58.2-76.5)	<.001	.23
Respiratory rate, breaths per minute	16.0 (14.6-17.6)	16.2 (14.3-17.9)	16.4 (14.5-18.6)	.31	<.001
Oxygen saturation, %	96.0 (94.5-97.5)	95.9 (94.4-97.6)	96.2 (94.8-97.8)	.28	<.001
Temperature, °C	37.2 (37.1-37.4)	37.3 (37.1-37.5)	37.2 (37.0-37.5)	.07	<.001
GCS eye opening response					
Median (IQR)	4.0 (4.0-4.0)	4.0 (4.0-4.0)	4.0 (4.0-4.0)	.42	<.001
<4	1058 (9.6)	133 (10.2)	398 (16.2)	.49	<.001
Surveillance during ward admission					
Continuous pulse oximetry	9408 (85.2)	1094 (83.8)	NA	.74	NA
Cardiac telemetry	460 (4.2)	225 (17.2)	NA	<.001	NA
DNR order status					
Ordered before first surgical procedure	55 (0.5)	53 (4.1)	27 (1.1)	<.001	<.001
Ordered during admission	78 (0.7)	91 (7.0)	59 (2.4)	<.001	<.001
Canceled during admission	6 (0.1)	18 (1.4)	15 (0.6)	<.001	.03

Abbreviations: bpm, beats per minute; DNR, do not resuscitate; GCS, Glasgow coma scale; ICU, intensive care unit; NA, not applicable.

^a^ *P* values are from significance tests comparing appropriate triage, undertriage, and control groups by each perioperative factor.

^b^ Postoperative variables, including postoperative vital signs, were not included as features in the estimation models used for cohort derivation.

^c^ Within 4 hours after surgical procedure end time.

The undertriaged group, compared with the control group, had similar immediate postoperative diastolic blood pressure but decreased median (IQR) systolic blood pressure (vs 131.0 [116.4-145.4] mm Hg; *P* < .001), respiratory rate (16.2 breaths per minute [14.3-17.9 breaths per minute] vs 16.4 breaths per minute [14.5-18.6 breaths per minute]; *P* < .001), and oxygen saturation (95.9% [94.4%-97.6%] vs 96.2% [94.8%-97.8%]; *P* < .001) and increased median (IQR) heart rate (82.1 bpm [72.2-91.4 bpm] vs 80.5 bpm [70.2-91.2 bpm], *P* = .01) and temperature (37.3 °C [37.1 °C-37.5 °C] vs 37.2 °C [37.0 °C-37.5 °C ]; *P* < .001). A decreased proportion of admitted patients who were undertriaged had a Glasgow coma scale eye opening response score less than 4 (133 admissions [10.2%] vs 398 admissions [16.2%]; *P* < .001) compared with admitted patients in the control group. An increased proportion of admitted patients who were undertriaged had a DNR order placed preoperatively (vs 27 admissions [1.1%]; *P* < .001) and during admission (vs 59 admissions [2.4%]; *P* < .001).

### Patient Outcomes

Patient outcomes are summarized in Table 3. The undertriaged group, compared with the appropriately triaged group, had increased incidence of ICU transfer (207 admissions [15.8%] vs 298 admissions [2.7%]; *P* < .001) at later median (IQR) postoperative times (71.0 [31.5-144.0] hours vs 35.0 [21.2-70.0] hours; *P* < .001). Undertriaged admissions had increased median (IQR) hospital length of stay (8.1 [5.1-13.6] days vs 3.0 [1.5-5.1] days; *P* < .001) and increased incidence of prolonged ICU admission (165 admissions [12.6%] vs 212 admissions [1.9%]; *P* < .001), prolonged mechanical ventilation (32 admissions [2.5%] vs 19 admissions [0.2%]; *P* < .001), postoperative red blood cell transfusion (325 admissions [24.9%] vs 475 admissions [4.3%]; *P* < .001), arterial catheter placement (173 admissions [13.2%] vs 370 admissions [3.4%]; *P* < .001), central venous catheter placement (37 admissions [2.8%] vs 49 admissions [0.4%]; *P* < .001), second surgical procedure during admission (360 admissions [27.6%] vs 536 admissions [4.9%]; *P* < .001), hospital mortality (19 admissions [1.5%] vs 5 admissions [0.05%]; *P* < .001), cardiac arrest (7 admissions [0.5%] vs 7 admissions [0.1%]; *P* < .001), unplanned intubation (45 admissions [3.4%] vs 48 admissions [0.4%]; *P* < .001), and acute kidney injury (341 admissions [26.1%] vs 965 admissions [8.7%]; *P* < .001) compared with appropriately triaged admissions. The undertriaged group had increased median (IQR) total charges per admission ($101 300 [$70 300-$155 000] vs $65 700 [$47 600-$88 300]; *P* < .001) and total costs per admission ($26 900 [$18 400-$42 300] vs $15 900 [$11 300-$22 500]; *P* < .001).

**Table 3.** Postoperative Outcomes

Outcome	Admissions, No (%)			*P* value^a^	
	Appropriate triage (n = 11 042)	Undertriage (n = 1306)	Control (n = 2452)	Appropriate triage vs undertriage	Undertriage vs control
Resource use					
Transfer from ward to ICU	298 (2.7)	207 (15.8)	NA	<.001	NA
Time from surgical procedures end to ICU transfer, median (IQR), hr	35.0 (21.2-70.0)	71.0 (31.5-144.0)	NA	<.001	NA
ICU admission ≥48 h	212 (1.9)	165 (12.6)	1407 (57.4)	<.001	<.001
Mechanical ventilation ≥48 h	19 (0.2)	32 (2.5)	53 (2.2)	<.001	.60
Postoperative red blood cell transfusion	396 (3.6)	275 (21.1)	337 (13.7)	<.001	<.001
Red blood cell transfusion during admission	475 (4.3)	325 (24.9)	380 (15.5)	<.001	<.001
Hospital length of stay, median (IQR), d	3.0 (1.5-5.1)	8.1 (5.1-13.6)	6.0 (3.3-9.3)	<.001	<.001
Postoperative procedure					
Arterial catheter	370 (3.4)	173 (13.2)	261 (10.6)	<.001	.02
Central venous catheter	49 (0.4)	37 (2.8)	51 (2.1)	<.001	.20
Bronchoscopy	9 (0.1)	8 (0.6)	23 (0.9)	<.001	.38
Chest tube	8 (0.1)	3 (0.2)	8 (0.3)	.13	.76
Electric cardioversion	4 (<0.1)	3 (0.2)	4 (0.2)	.04	.71
Second surgical procedure during admission	536 (4.9)	360 (27.6)	353 (14.4)	<.001	<.001
Emergent second surgical procedure	107 (1.0)	92 (7.0)	91 (3.7)	<.001	<.001
Time between surgical procedures, median (IQR), d	2.6 (1.8-4.7)	3.8 (2.0-6.0)	3.3 (1.6-6.3)	<.001	.23
Complication					
Hospital mortality	5 (0.1)	19 (1.5)	17 (0.7)	<.001	.04
Discharge to hospice	13 (0.1)	23 (1.8)	14 (0.6)	<.001	<.001
Cardiac arrest	7 (0.1)	7 (0.5)	10 (0.4)	<.001	.64
Unplanned intubation	48 (0.4)	45 (3.4)	49 (2.0)	<.001	.01
Acute kidney injury	965 (8.7)	341 (26.1)	477 (19.5)	<.001	<.001
Rapid reversal	459 (4.2)	153 (11.7)	248 (10.1)	<.001	.16
Persistent					
With kidney recovery	100 (0.9)	97 (7.4)	91 (3.7)	<.001	<.001
Without kidney recovery	406 (3.7)	91 (7.0)	138 (5.6)	<.001	.14
Charges and costs, median (IQR), thousands of $					
Professional service charges	13.7 (10.0-18.5)	18.5 (13.1-30.2)	24.5 (16.9-37.9)	<.001	<.001
Charges for hospital admission	65.7 (47.6-88.3)	101.3 (70.3-155.0)	120.2 (84.5-163.7)	<.001	<.001
Costs for hospital admission	15.9 (11.3-22.5)	26.9 (18.4-42.3)	32.7 (22.7-48.5)	<.001	<.001

Abbreviations: ICU, intensive care unit; NA, not applicable.

^a^ *P* values were adjusted for multiple comparisons using the Benjamini-Hochberg procedure. *P* values are from significance tests comparing appropriate triage, undertriage, and control groups by each outcome.

The undertriaged group, compared with the control group, had decreased proportions of admitted patients with prolonged ICU admission (vs 1407 admissions [57.4%]; *P* < .001), similar proportions of admitted patients with prolonged mechanical ventilation (vs 53 admissions [2.2%]; *P* = .60), decreased proportions of admitted patients receiving postoperative red blood cell transfusion (275 admissions [21.1%] vs 337 admissions [13.7%]; *P* < .001), and increased median (IQR) hospital length of stay (vs 6.0 [3.3-9.3] days; *P* < .001). There was also an increased proportion of admitted patients who were undertriaged undergoing postoperative arterial catheter placement (vs 261 admissions [10.6%]; *P* = .02), second surgical procedure during admission (vs 353 admissions [14.4%]; *P* < .001), and emergent second surgical procedure (vs 91 admissions [3.7%]; *P* < .001) compared with admitted patients in the control group. Intervals between first and second surgical procedures were similar between undertriaged and control groups (median [IQR], 3.8 [2.0-6.0] days vs 3.3 [1.6-6.3] days; *P* = .23). The undertriaged group, compared with the control group, had increased incidence of hospital mortality (vs 17 admissions [0.7%]; *P* = .04), discharge to hospice (23 admissions [1.8%] vs 14 admissions [0.6%]; *P* < .001), unplanned intubation (vs 49 admissions [2.0%]; *P* = .01), and acute kidney injury (vs 477 admissions [19.5%]; *P* < .001), which was primarily associated with increased incidence of persistent acute kidney injury with kidney recovery (97 admissions [7.4%] vs 91 admissions [3.7%]; *P* < .001). Undertriaged admissions had decreased median (IQR) total charges per admission (vs $120 200 [$84 500-$163 700]; *P* < .001) and total costs per admission (vs $32 700 [$22 700-$48 500]; *P* < .001) compared with the control group.

### Secondary Analyses

Exploratory secondary analyses investigating whether there were associations between self-identification as Black or African American, area deprivation indices, and preoperative DNR orders and outcomes are illustrated in eFigure 2, eFigure 3, and eFigure 4 in the Supplement, respectively; we provide analytic details in figure legends. Appropriately triaged admissions were excluded from these analyses owing to substantial differences in baseline characteristics and outcomes. Secondary analysis outcomes were hospital mortality or discharge to hospice, unplanned intubation, and acute kidney injury because these outcomes differed between undertriaged and control groups in primary analyses.

There were no associations of area deprivation indices or self-identification as Black or African American with selected outcomes. Admitted patients with preoperative DNR orders in the undertriaged and control groups had increased incidence of hospital death or discharge to hospice (5 admissions [6.2%] vs 68 admissions [1.8%]; *P* = .02) and acute kidney injury (32 admissions [40.0%] vs 786 admissions [21.4%]; *P* < .001) compared with admitted patients without DNR orders. The undertriaged group had an increased proportion of admitted patients with both preoperative DNR orders and postoperative acute kidney injury (19 admissions [1.5%] vs 13 admissions [0.5%]; *P* = .005) compared with the control group; the proportion of admitted patients with both preoperative DNR orders and death or hospice were similar between the undertriage and control groups (3 admissions [0.2%] vs 2 admissions [0.1%]; *P* = .35). There were no associations between preoperative DNR orders and unplanned intubation.

## Discussion

This cross-sectional study found that approximately 11% of postoperative ward admissions had increased risk of postoperative complications and had increased short-term mortality and morbidity compared with risk-matched ICU admissions. The observed increased incidence of preoperative DNR orders among undertriaged admissions may have been associated with decreased escalation of care in response to impending or evolving postoperative complications; deeper analyses of palliative care consultations and clinical notes are needed to address this hypothesis. Undertriaged admissions had increased incidence of cardiac telemetry; among patients admitted to wards who were at increased risk of complications, continuous monitoring may be associated with increased early detection of decompensation without requiring ICU resources.^15,16,17^ Additionally, we found that patients who were at increased risk of postoperative complications and undertriaged were accurately identified using automated EHR data from preoperative and intraoperative phases of care, suggesting opportunities for data-driven decision support. These results could be operationalized by generating EHR advisories triggered by ward admission orders placed for patients who have received surgical procedures who are at increased risk of complications.

Previous work has characterized postoperative triage patterns by classifying patients at increased risk of complications using a single parameter: high-risk procedures with mortality rates of 5% or greater. Using this approach in a National Health Service study, Pearse et al^2^ provided a sentinel report that patients undergoing high-risk surgical procedures accounted for 13% of surgical procedures and 84% of deaths. Notably, fewer than 15% of patients at increased risk of complications were triaged to ICUs postoperatively. In a subsequent study by Jhanji et al,^3^ 35% of patients at increased risk of complications were admitted to an ICU at any point after their surgical procedures. Overall, the high-risk mortality rate was 12%, but among patients at increased risk of complications who were initially triaged to a ward and subsequently transferred to the ICU, mortality was 30%. Others have assessed the ability of physicians to estimate adverse events when making triage decisions. In a multicenter study of patients admitted to ICUs for medical and surgical procedures who were transferred to hospital wards, physicians surveyed at the time of ICU discharge exhibited mediocre performance in estimating adverse events, ICU readmissions, and hospital mortality.^18^ A similar phenomenon may occur at surgical procedure end time, although further investigation is needed to address this hypothesis.

The methods presented in our study are unique, which hinders direct comparisons with previous work, but our results are generally consistent with those in peer-reviewed literature. Advanced age, demographic variables, comorbidities, American Society of Anesthesiologists physical status classifications, and organ dysfunction have all been previously described as major risk factors associated with postoperative mortality and morbidity.^9,10,19,20,21,22,23,24,25,26,27,28^ Analyses including area deprivation indices in postoperative outcomes are sparse, but Carmichael et al^29^ found that increased area deprivation indices, along with other indicators associated with social vulnerability, were associated with increased odds of undergoing emergency surgical procedures. We observed increased area deprivation indices among undertriaged admissions, which may be associated with overlap with emergency admission priority and surgical procedure priority or an odious association with biased care or unobserved disease; further investigation of these potential etiologies is needed. We observed that DNR orders were associated with increased incidence of postoperative mortality, as was previously described by Kazaure et al.^30^ Overall, although our postoperative acuity assessment methods are unique, results based on our methods are similar to results from similar studies.

### Limitations

This study has several limitations. Our study was limited by its single institution design and may not be generalizable to other practice settings. In addition, the retrospective analysis of EHR data is associated with risk for selection bias. We sought to minimize selection bias by including all consecutive admissions meeting inclusion criteria. These analyses excluded decisions for postoperative admission vs discharge home (ie, ambulatory surgical procedures), which require further investigation. Time-splitting development and validation cohorts allow bias from practice changes over time but may also mitigate the potential for model performance degradation from data set drift when applied prospectively.^11^ Although model discrimination was strong, precision in estimating hospital mortality was poor, as is often observed when estimating rare outcomes. Additionally, our power analysis was targeted loosely, owing to the lack of similar studies in peer-reviewed literature. This underscores a greater problem: despite the severe consequences associated with postoperative undertriage, there is sparse evidence regarding this topic, which hinders the development of decision-support tools. In the absence of efficient, effective decision-support platforms to augment postoperative triage decisions, surgeons must rely on individual judgement and hypothetical-deductive reasoning, which is highly variable and error-prone and may contribute to variability in surgical care costs and the incidence of failure to rescue across US hospitals.^31,32^

## Conclusions

This study found that patients at increased risk for postoperative complications who underwent surgical procedures and were undertriaged to hospital wards had increased mortality and morbidity compared with patients in a risk-matched control group admitted to ICUs. Postoperative undertriage was identifiable using automated preoperative and intraoperative EHR data as features in machine-learning models that made data-driven, patient-level risk assessments. These findings may provide a framework and rationale for real-time clinical decision support platforms to augment postoperative triage decisions.

## Notes

Supplement. **eFigure 1.** Derivation of Study Population **eTable 1.** Primary Surgical Service **eTable 2.** Hospital Mortality Rates for Each Primary Surgical Service **eTable 3.** 20 Most Important Features for Estimating Hospital Mortality **eTable 4.** 20 Most Important Features for Estimating Prolonged Intensive Care Unit Stay **eFigure 2.** Subgroup Analyses of Associations Between Outcomes and Self-identification as Black or African American **eFigure 3.** Subgroup Analyses of Associations Between Outcomes and Area Deprivation Index (ADI) National Ranks **eFigure 4.** Subgroup Analyses of Associations Between Outcomes and Preoperative Do No Resuscitate (DNR) Orders
eMethods.
