# Supplementary material for: Association of Postoperative Undertriage to Hospital Wards With Mortality and Morbidity
Source: JAMA Netw Open. 2021 Nov 10;4(11):e2131669. doi: 10.1001/jamanetworkopen.2021.31669 (PMC8581722; doi:10.1001/jamanetworkopen.2021.31669)
Supplement: Supplement. — eFigure 1. Derivation of Study Population eTable 1. Primary Surgical Service eTable 2. Hospital Mortality Rates for Each Primary Surgical Service eTable 3. 20 Most Important Features for Estimating Hospital Mortality eTable 4. 20 Most Important Features for Estimating Prolonged Intensive Care Unit Stay eFigure 2. Subgroup Analyses of Associations Between Outcomes and Self-identification as Black or African American eFigure 3. Subgroup Analyses of Associations Between Outcomes and Area Deprivation Index (ADI) National Ranks eFigure 4. Subgroup Analyses of Associations Between Outcomes and Preoperative Do No Resuscitate (DNR) Orders eMethods. [file jamanetwopen-e2131669-s001.pdf]

Supplemental Online Content

Loftus TJ, Ruppert MM, Ozrazgat-Baslanti T, et al. Association of postoperative undertriage to hospital wards with mortality and morbidity. *JAMA Netw Open*. 2021;4(11):e2131669. doi:10.1001/jamanetworkopen.2021.31669

eFigure 1. Derivation of Study Population
eTable 1. Primary Surgical Service
eTable 2. Hospital Mortality Rates for Each Primary Surgical Service
eTable 3. 20 Most Important Features for Estimating Hospital Mortality
eTable 4. 20 Most Important Features for Estimating Prolonged Intensive Care Unit Stay
eFigure 2. Subgroup Analyses of Associations Between Outcomes and Self-Identification as Black or African American
eFigure 3. Subgroup Analyses of Associations Between Outcomes and Area Deprivation Index (ADI) National Ranks
eFigure 4. Subgroup Analyses of Associations Between Outcomes and Preoperative Do No Resuscitate (DNR) Orders
eMethods.

This supplemental material has been provided by the authors to give readers additional information about their work.

© 2021 Loftus TJ et al. *JAMA Network Open.*

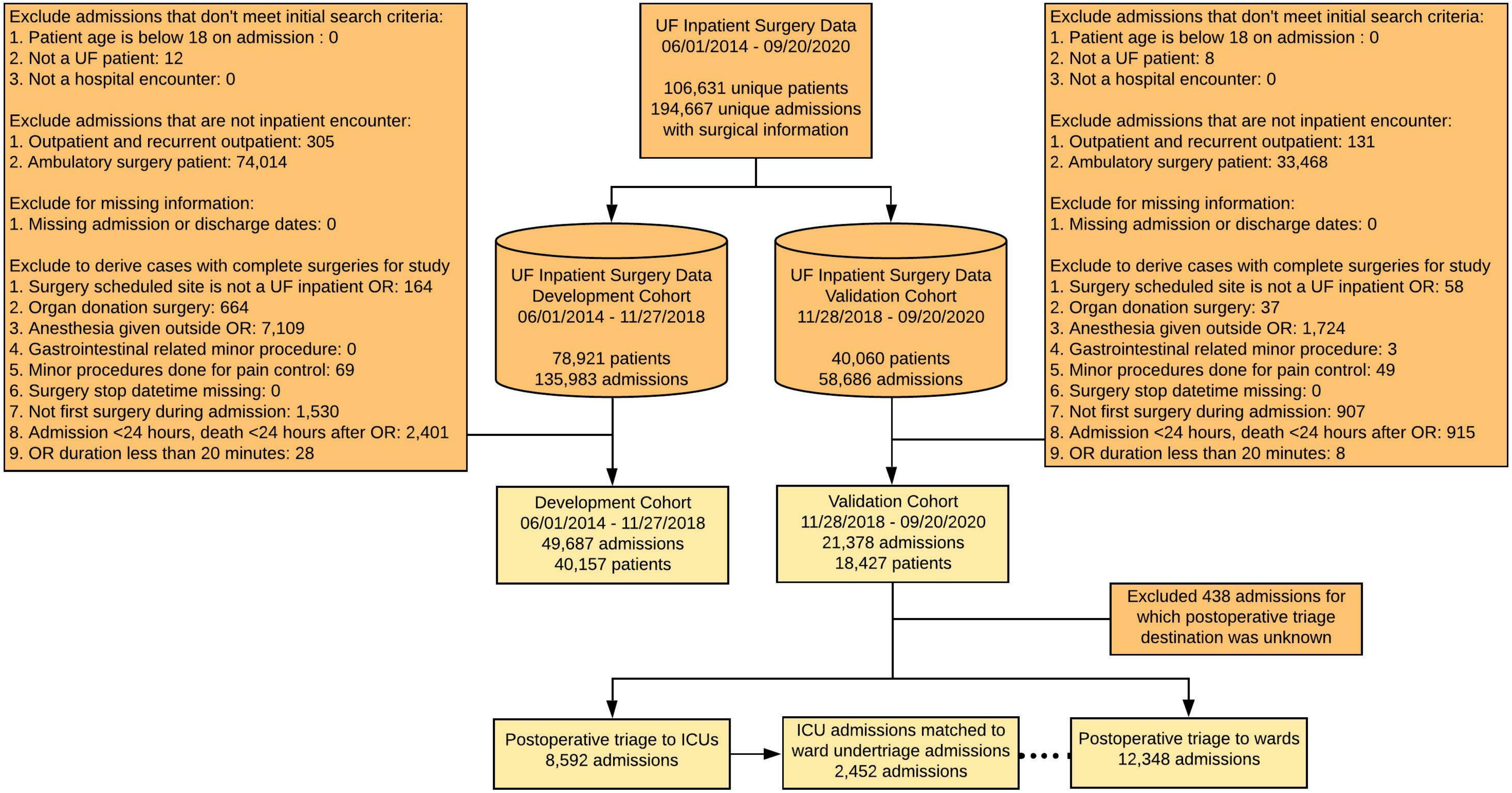

eFigure 1. Derivation of Study Population

UF: University of Florida, OR: operating room, ICU: intensive care unit.

© 2021 Loftus TJ et al. *JAMA Network Open.*

eTable 1. Primary Surgical Service

**Primary surgical service**	**Appropriate triage** N=11,042 No (%)	**Undertriage** N=1,306 No (%)	**Controls** N=2,452 No (%)	**P[a]**	**P[b]**
Breast, melanoma, sarcoma, endocrine	130 (1.2)	21 (1.6)	9 (0.4)	.18	**<.001**
Burn surgery	80 (0.7)	10 (0.8)	75 (3.1)	.86	**<.001**
Colorectal surgery	443 (4.0)	36 (2.8)	34 (1.4)	**.03**	**.005**
Ear, nose, and throat surgery	287 (2.6)	47 (3.6)	241 (9.8)	**.04**	**<.001**
Gynecologic surgery	539 (4.9)	20 (1.5)	15 (0.6)	**<.001**	**.007**
Minimally invasive surgery	552 (5.0)	21 (1.6)	15 (0.6)	**<.001**	**.004**
Neurosurgery	1,253 (11.3)	105 (8.0)	696 (28.4)	**<.001**	**<.001**
Ophthalmology	50 (0.5)	28 (2.1)	9 (0.4)	**<.001**	**<.001**
Oral surgery	134 (1.2)	10 (0.8)	17 (0.7)	.17	.84
Orthopedic surgery	4,398 (39.8)	302 (23.1)	121 (4.9)	**<.001**	**<.001**
Pancreas and biliary surgery	73 (0.7)	19 (1.5)	79 (3.2)	**.004**	**.001**
Pediatric surgery (age 18 years or greater)	73 (0.7)	14 (1.1)	4 (0.2)	.11	**<.001**
Podiatry	22 (0.2)	14 (1.1)	1 (0.0)	**<.001**	**<.001**
Plastic and reconstructive surgery	223 (2.0)	46 (3.5)	57 (2.3)	**<.001**	**.04**
Thoracic and cardiovascular surgery	60 (0.5)	47 (3.6)	410 (16.7)	**<.001**	**<.001**
Transplant surgery (liver, kidney)	218 (2.0)	45 (3.4)	28 (1.1)	**.001**	**<.001**
Trauma and acute care surgery	873 (7.9)	145 (11.1)	151 (6.2)	**<.001**	**<.001**
Urology	1,125 (10.2)	69 (5.3)	74 (3.0)	**<.001**	**<.001**
Vascular surgery	509 (4.6)	307 (23.5)	416 (17.0)	**<.001**	**<.001**

P values correspond to significance tests comparing the appropriate triage, undertriage, and control cohorts by each variable listed in the “Primary surgical service” column.

eTable 2. Hospital Mortality Rates for Each Primary Surgical Service

**Primary surgical service**	**Hospital mortality** No (%)
Breast, melanoma, sarcoma, endocrine	0 (0.0)
Burn surgery	2 (4.9)
Colorectal surgery	2 (4.9)
Ear, nose, and throat surgery	2 (4.9)
Gynecologic surgery	2 (4.9)
Minimally invasive surgery	1 (2.4)
Neurosurgery	9 (22.0)
Ophthalmology	1 (2.4)
Oral surgery	0 (0.0)
Orthopedic surgery	6 (14.6)
Pancreas and biliary surgery	0 (0.0)
Pediatric surgery (age 18 years or greater)	0 (0.0)
Podiatry	0 (0.0)
Plastic and reconstructive surgery	0 (0.0)
Thoracic and cardiovascular surgery	1 (2.4)
Transplant surgery (liver, kidney)	0 (0.0)
Trauma and acute care surgery	6 (14.6)
Urology	4 (9.8)
Vascular surgery	5 (12.2)

© 2021 Loftus TJ et al. *JAMA Network Open.*

eTable 3. 20 Most Important Features for Estimating Hospital Mortality

**Features**	**Weight**
Primary procedure	0.039
Median intraoperative minimum alveolar concentration[a]	0.031
Mean intraoperative minimum alveolar concentration[a]	0.030
Scheduled postoperative location	0.018
Minimum intraoperative fraction of inspired oxygen	0.017
Attending surgeon	0.014
Mean intraoperative fraction of inspired oxygen	0.012
Maximum international normalize ratio within one week before surgery	0.011
Mean international normalize ratio within one week before surgery	0.011
Duration of measuring minimum alveolar concentration[a] intraoperatively	0.011
Number of serum lactate measurements within one week before surgery	0.011
Indicator for missing positive end-expiratory pressure values	0.010
Percentage of intraoperative fraction of inspired oxygen <40%	0.010
Surgery type	0.010
Minimum serum albumin within one week before surgery	0.008
Number of intraoperative fraction of inspired oxygen <40%	0.008
Scheduled operating room	0.008
Having a serum pH measured within one week before surgery	0.008
Mean serum albumin within one week before surgery	0.008
Maximum blood urea nitrogen within one week before surgery	0.007

[a]Age adjusted.

© 2021 Loftus TJ et al. *JAMA Network Open.*

eTable 4. 20 Most Important Features for Estimating Prolonged Intensive Care Unit Stay

Features	Weight
Scheduled postoperative location	0.251
Primary procedure	0.146
Duration of intraoperative inhalational anesthetic administration	0.125
Attending surgeon	0.050
Surgery type	0.029
Number of intraoperative mean arterial pressures in the range 68-88 mmHg mmHg	0.026
Number of intraoperative diastolic pressures in the range 53-71 mmHg mmHg	0.026
Indicator for missing positive end-expiratory pressure values	0.020
Number of intraoperative systolic pressures in the range 99-127 mmHg mmHg	0.013
Median intraoperative minimum alveolar concentration[a]	0.010
Minimum intraoperative fraction of inspired oxygen	0.010
Mean intraoperative minimum alveolar concentration[a]	0.009
More than 107 heart rate measurements intraoperatively	0.008
Number of serum lactate measurements within one week before surgery	0.008
Scheduled operating room	0.008
Number of intraoperative mean arterial pressures in the range 73-83 mmHg mmHg	0.007
Number of serum glucose measurements within one week before surgery	0.007
Having a serum pH measured within one week before surgery	0.007
Number of intraoperative diastolic pressures in the range 44-58 mmHg mmHg	0.006
Number of hemoglobin measurements within one week before surgery	0.006

[a]Age adjusted.

© 2021 Loftus TJ et al. *JAMA Network Open.*

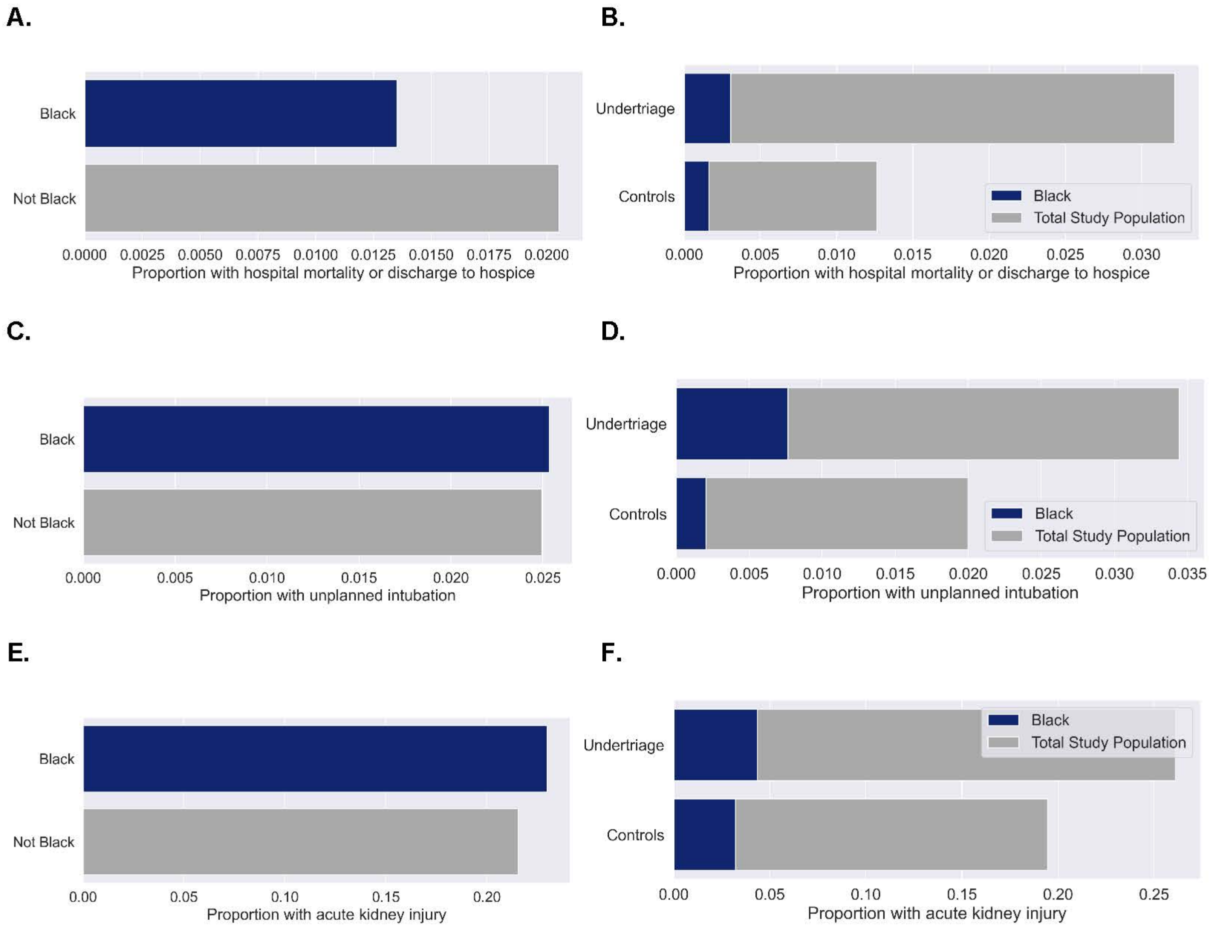

Supplemental eFigure 2. Subgroup analyses of associations between outcomes and self-identification as Black or African American. Proportions were compared by Fisher's Exact test. The total study population for these exploratory, secondary analyses represents all postoperative admissions that were undertriaged to hospital wards or risk-matched control admissions to intensive care units. **A.** The incidence of hospital mortality or discharge to hospice was similar between admissions in which the patient self-identified as Black or African American and admissions in which the patient did not. **B.** The incidence of hospital mortality or discharge to hospice was similar between undertriaged admissions in which the patient self-identified as Black or African American and control admissions in which the patient self-identified as Black or African American. **C.** The incidence of unplanned intubation was similar between admissions in which the patient self-identified as Black or African American and admissions in which the patient did not. **D.** The incidence of unplanned intubation was similar between undertriaged admissions in which the patient self-identified as Black or African American and control admissions in which the patient self-identified as Black or African American. **E.** The incidence of acute kidney injury was similar between admissions in which the patient self-identified as Black or African American and admissions in which the patient did not. **E.** The incidence of acute kidney injury was similar between undertriaged admissions in which the patient self-identified as Black or African American and control admissions in which the patient self-identified as Black or African American.

eFigure 2. Subgroup Analyses of Associations Between Outcomes and Self-Identification as Black or African American

© 2021 Loftus TJ et al. *JAMA Network Open.*

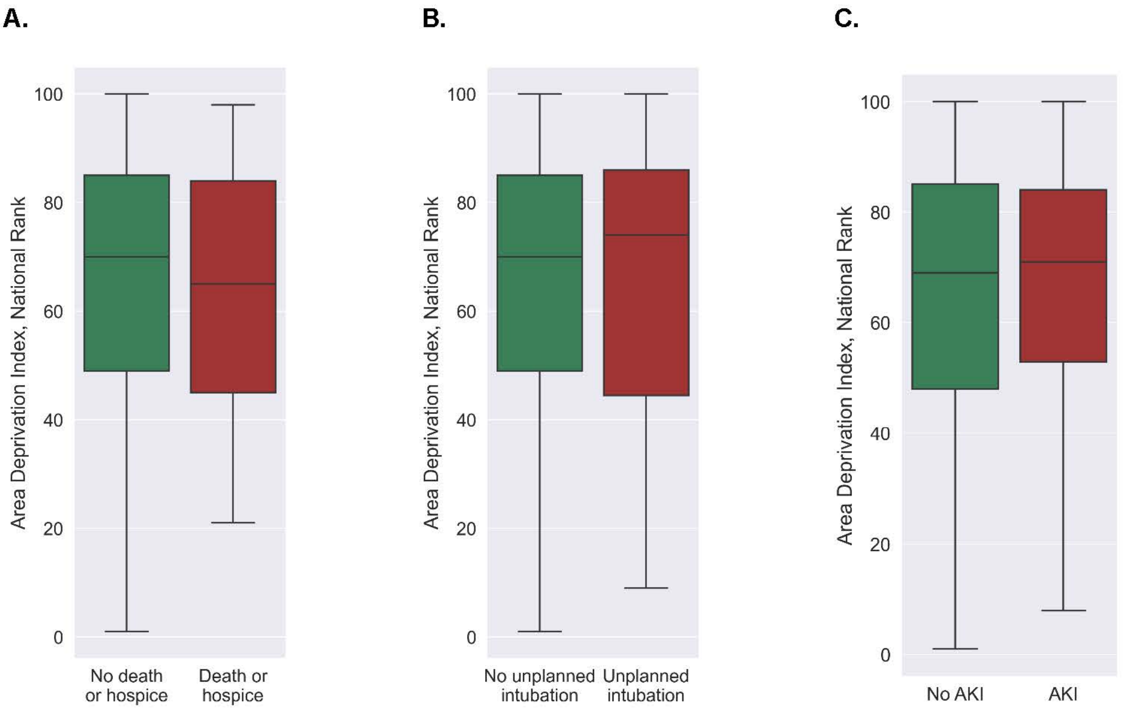

Supplemental eFigure 3. Subgroup analyses of associations between outcomes and area deprivation index (ADI) national ranks. Data are presented as median values, boxes representing interquartile ranges, and whiskers representing 1.5 times the interquartile range. ADI values were compared by the Kruskal-Wallis test. **A.** ADI national ranks had similar distributions for admissions with and without death or hospice. **B.** ADI national ranks had similar distributions for admissions with and without unplanned intubation. **C.** ADI national ranks had similar distributions for admissions with and without acute kidney injury (AKI).

eFigure 3. Subgroup Analyses of Associations Between Outcomes and Area Deprivation Index (ADI) National Ranks

© 2021 Loftus TJ et al. *JAMA Network Open.*

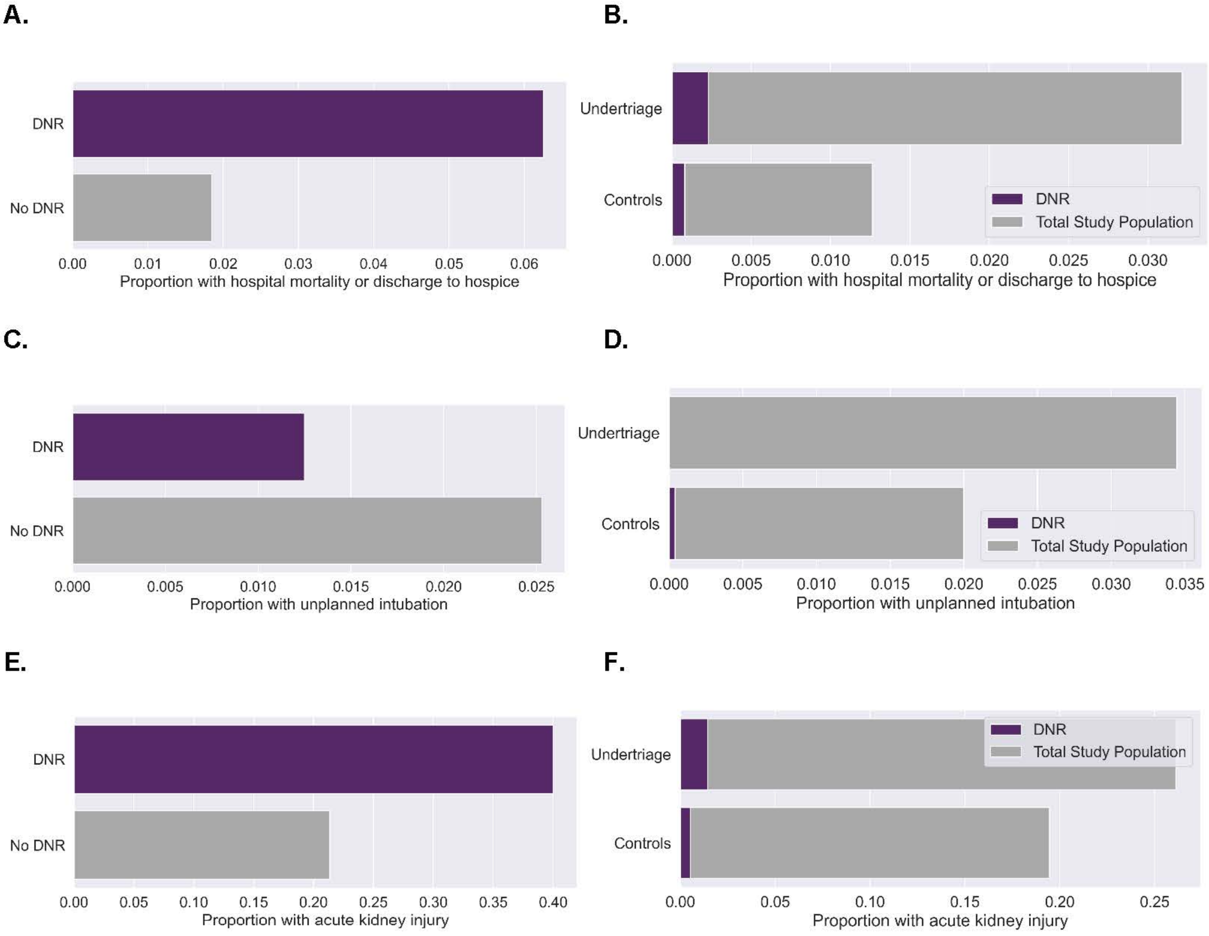

Supplemental eFigure 4. Subgroup analyses of associations between outcomes and preoperative Do Not Resuscitate (DNR) orders. Proportions were compared by Fisher's Exact test. The total study population for these exploratory, secondary analyses represents all postoperative admissions that were undertriaged to hospital wards or risk-matched control admissions to intensive care units. **A.** Admissions with a preoperative DNR order had greater incidence of hospital death or discharge to hospice (6.2% vs. 1.8%, P=.02). **B.** The incidence of hospital mortality or discharge to hospice was similar between undertriaged admissions with a DNR order and control admissions with a DNR order (0.2% vs. 0.1%, respectively, P=.35). **C.** The incidence of unplanned intubation was similar between admissions with a DNR order and those without a DNR order (1.2% vs. 2.5%, respectively, P=0.72). **D.** The incidence of unplanned intubation was similar between undertriaged admissions with a DNR order and control admissions with a DNR order. **E.** Admissions with a preoperative DNR order had greater incidence of postoperative acute kidney injury (40.0% vs. 21.4%, P<.001). **F.** Compared with controls, the undertriage cohort had a greater proportion with both preoperative DNR order and postoperative acute kidney injury (1.5% vs. 0.5%, P=.005).

eFigure 4. Subgroup Analyses of Associations Between Outcomes and Preoperative Do No Resuscitate (DNR) Orders

© 2021 Loftus TJ et al. *JAMA Network Open.*

eMethods.

Overview of Data Elements

The final dataset included granular information from preoperative, intraoperative, and postoperative phases of care regarding demographics, socioeconomic status approximated by the area deprivation index state and national ranks, Do Not Resuscitate orders and cancellations, diagnoses, operating room and postoperative procedures, vital signs, laboratory values, medications, blood product transfusions, movements among hospital stations, complications (i.e., hospital mortality, cardiac arrest, unplanned intubation, and acute kidney injury), lengths of stay in the hospital and intensive care unit (ICU), duration of mechanical ventilation, costs for the hospital admission, and charges billed by providers and for the hospital admission.[12]

Data Elements for Model Development and Validation

Data obtained from electronic health records for generating predictions for hospital mortality and prolonged ICU stay included demographics (including race and ethnicity), insurance and socio-economic features, preoperative comorbidities derived from International Classification of Diseases codes, laboratory values and vital signs including intraoperative phases of care, surgery type, medication orders derived from RxNorm codes grouped into drug classes, emergency department admission, admission priority, surgery priority, and intraoperative physiologic data including heart rate, systolic blood pressure, diastolic blood pressure, body temperature, respiratory rate, minimum alveolar concentration, positive end-expiratory pressure, peak inspiratory pressure, fraction of inspired oxygen, blood oxygen saturation, and end-tidal carbon dioxide.[13,14] Charlson comorbidity index scores were used in risk predictions and were calculated based exclusively on comorbid conditions that were coded prior to admission or identified as being present on admission.

Data Elements for Clinical Outcomes

Outcomes representing resource use included subsequent transfers from wards to ICUs, ICU length of stay, prolonged (48 hours or greater) mechanical ventilation, postoperative red cell transfusions, total red cell transfusions during admission, hospital length of stay, professional service charges (i.e., dollars billed by providers during admission), total charges (i.e., dollars billed by the hospital during admission), and total costs (i.e., costs incurred by the hospital during admission). Postoperative procedures included arterial catheter placement, central venous catheter placement, bronchoscopy, chest tube insertion, and electric cardioversion. Complications included the following adverse events:

© 2021 Loftus TJ et al. *JAMA Network Open.*

hospital mortality, cardiac arrest, unplanned intubation, and acute kidney injury. Cardiac arrest was defined as resuscitation requiring chest compressions or defibrillation.[16] Unplanned intubation was defined as any intubation occurring outside of the operating room. Acute kidney injury was classified as rapidly reversed within 48 hours, persistent beyond 48 hours but recovered at the time of hospital discharge, or persistent and not recovered at the time of hospital discharge, consistent with previous work in surgical patients.[17]

Summary of Model Input Features

Variable	Type	Number of categories	Preprocessing steps
**Demographic Variables**			
Age (years)	Continuous		Outlier imputation[a]
Gender	Binary	2	
Race	Nominal	4	Categorical feature optimization[b]
Body Mass Index	Continuous		Outlier imputation[a]
Marital Status	Nominal	3	Categorical feature optimization[b]
Ethnicity	Binary	2	
**Socioeconomic Variables**			
Primary Insurance	Nominal	4	Categorical feature optimization[b]
Residency area characteristics			
Zip code	Nominal	1,908	Transformation through link to Census data[c]
Rural area	Binary	2	
Total Population	Continuous		Obtained using residency zip code with linkage to US Census data[c]; Outlier imputation[a]
Median Income	Continuous		Obtained using residency zip code with linkage to US Census data[c]; Outlier imputation[a]
Proportion of African Americans	Continuous		Obtained using residency zip code with linkage to US Census data[c]; Outlier imputation[a]
Proportion of Hispanics	Continuous		Obtained using residency zip code with linkage to US Census data[c]; Outlier imputation[a]
Proportion Below Poverty	Continuous		Obtained using residency zip code with linkage to US Census data[c]; Outlier imputation[a]
Distance from Residency to Hospital (km)	Continuous		Obtained using residency zip code with linkage to US Census data[c]; Outlier imputation[a]
**Operative Characteristics**			
Day of admission	Nominal	7	Categorical feature optimization[b]
Month of admission	Nominal	12	Categorical feature optimization[b]
Attending Surgeon	Nominal	324	Categorical feature optimization[b]
Admission Source	Binary	2	

© 2021 Loftus TJ et al. *JAMA Network Open.*

Variable	Type	Number of categories	Preprocessing steps
Admission Type (Emergent/Elective)	Binary	2	
Admitting Type (Medicine/Surgery)	Binary	2	
Admitting Service	Nominal	46	Categorical feature optimization[b]
Night Admission	Binary	2	
Scheduled Surgery Type	Nominal	15	Categorical feature optimization[b]
Scheduled Surgery Room	Nominal	41	Categorical feature optimization[b]
Scheduled post operation location	Binary	2	
Scheduled room is trauma room	Binary	2	
Time of surgery from admission (days)	Continuous		Outlier imputation[a]
Scheduled primary surgical procedure	Nominal	2,142	Forest tree analysis of ICD9 codes[d]
**Comorbidities**			
Charlson Comorbidity Index	Nominal	18	Categorical feature optimization[b]
Myocardial Infarction	Binary	2	
Congestive Heart Failure	Binary	2	
Peripheral Vascular Disease	Binary	2	
Cerebrovascular Disease	Binary	2	
Chronic Pulmonary Disease	Binary	2	
Diabetes	Binary	2	
Cancer	Binary	2	
Liver Disease	Binary	2	
Valvular disease	Binary	2	
Coagulopathy	Binary	2	
Weight loss	Binary	2	
Alcohol or Drug Abuse	Binary	2	
Smoking Status	Nominal	4	Categorical feature optimization[b]
**Medications History[e]**			
Betablockers	Binary	2	
Diuretics	Binary	2	
Statin	Binary	2	
Aspirin	Binary	2	
Angiotensin-Converting-Enzyme Inhibitors	Binary	2	
Pressors or Inotropes	Binary	2	
Bicarbonate	Binary	2	
Antiemetic	Binary	2	
Aminoglycosides	Binary	2	
Vancomycin	Binary	2	
Nonsteroidal Anti-inflammatory Drug	Binary	2	
**Preoperative Laboratory Results**			
Urine Protein, mg/dL	Nominal	5	Categorical feature optimization[b]
Urine Hemoglobin, mg/dL	Nominal	5	Categorical feature optimization[b]
Urine Glucose, mg/dL	Nominal	5	Categorical feature optimization[b]
Urine Erythrocytes, mg/dL	Nominal	5	Categorical feature optimization[b]
Serum Glucose, mg/dL	Continuous		Outlier imputation[a]
Blood Urea Nitrogen test, mg/dL	Continuous		Outlier imputation[a]

© 2021 Loftus TJ et al. *JAMA Network Open.*

Variable	Type	Number of categories	Preprocessing steps
Serum Creatinine, mg/dL	Continuous		Outlier imputation[a]
Serum Calcium, mmol/L	Continuous		Outlier imputation[a]
Serum Sodium, mmol/L	Continuous		Outlier imputation[a]
Serum Potassium, mmol/L	Continuous		Outlier imputation[a]
Serum Chloride, mmol/L	Continuous		Outlier imputation[a]
Serum CO2, mmol/L	Continuous		Outlier imputation[a]
Serum White Blood Cell, thou/uL	Continuous		Outlier imputation[a]
Mean Corpuscular Hemoglobin in Blood, g/dL	Continuous		Outlier imputation[a]
Mean Corpuscular Hemoglobin Concentration in Blood, pg	Continuous		Outlier imputation[a]
Erythrocyte Distribution Width Count, %	Continuous		Outlier imputation[a]
Serum creatinine, mg/dL	Continuous		Outlier imputation[a]
Serum Platelet, thou/uL	Continuous		Outlier imputation[a]
Serum Hemoglobin, g/dL	Continuous		Outlier imputation[a]
Reference Estimated Glomerular Filtration Rate, mL/min/1.73 m²	Continuous		Outlier imputation[a]
Urea nitrogen-Creatinine ratio in Serum	Continuous		Outlier imputation[a]
**Physiologic Intraoperative Time Series**			
Systolic blood pressure, mmHg	Continuous		Data cleaning[f]; Outlier imputation[g]; Statistical features extraction[h]
Diastolic blood pressure, mmHg	Continuous		Data cleaning[f]; Outlier imputation[g]; Statistical features extraction[h]
Minimum alveolar concentration	Continuous		Data cleaning[f]; Outlier imputation[g]; Statistical features extraction[h]
Heart rate, bpm	Continuous		Data cleaning[f]; Outlier imputation[g]; Statistical features extraction[h]
Temperature (°C)			
Peripheral capillary oxygen saturation (SPO2)	Continuous		Data cleaning[f]; Outlier imputation[g]; Statistical features extraction[h]
End-tidal CO2 (ETCO2)	Continuous		Data cleaning[f]; Outlier imputation[g]; Statistical features extraction[h]
Peak Inspiratory Pressure (PIP)	Continuous		Data cleaning[f]; Outlier imputation[g]; Statistical features extraction[h]
Positive End-expiratory Pressure (PEEP)	Continuous		Data cleaning[f]; Outlier imputation[g]; Statistical features extraction[h]
Respiratory O2	Continuous		Data cleaning[f]; Outlier imputation[g]; Statistical features extraction[h]
Respiratory Rate	Continuous		Data cleaning[f]; Outlier imputation[g]; Statistical features extraction[h]
**Laboratory Results from Surgery**			
Fraction of inspired oxygen (FIO2)	Continuous		Data cleaning[f]; Outlier imputation[g]; Statistical features extraction[h]
**Other Characteristics**			
Duration of surgery, min	Continuous		
Estimated blood loss, mL	Continuous		Missing value imputed by 0
Urine output, mL	Continuous		Missing value imputed by 0

© 2021 Loftus TJ et al. *JAMA Network Open.*

[a]For continuous variables, observations within in the top and bottom 1% of the distribution were considered outliers.
[b]For categorical variables with more than two levels, levels were transformed to numeric values.
[c]Using residency zip code, we linked to US Census data to calculate residing neighborhood characteristics and distance from hospital.
[d]Surgical procedure codes were optimized using forest tree analysis of ICD-9-CM codes.
[e]Medications within one year prior to surgery using RxNorms data grouped into drug classes according to the US, Department of Veterans Affairs National Drug File-Reference Terminology.
[f]We used observations from the first surgery during admission.
[g]Values outside of the predefined ranges were removed.
[h]We extracted several descriptive statistical measures, i.e., mean and standard deviation of time series, minimum and maximum values observed, and raw time and percentage of time a patient spent in a specific range of values for each of the time series.

© 2021 Loftus TJ et al. *JAMA Network Open.*
